# Connexins and Diabetes

**DOI:** 10.1155/2012/496904

**Published:** 2012-03-01

**Authors:** Josephine A. Wright, Toby Richards, David L. Becker

**Affiliations:** Department of Vascular Surgery and Department of Cell and Developmental Biology, University College London Hospital, 74 Huntley Street, London NW1 2BU, UK

## Abstract

Cell-to-cell interactions via gap junctional communication and connexon hemichannels are involved in the pathogenesis of diabetes. Gap junctions are highly specialized transmembrane structures that are formed by connexon hemichannels, which are further assembled from proteins called “connexins.” In this paper, we discuss current knowledge about connexins in diabetes. We also discuss mechanisms of connexin influence and the role of individual connexins in various tissues and how these are affected in diabetes. Connexins may be a future target by both genetic and pharmacological approaches to develop treatments for the treatment of diabetes and its complications.

## 1. Introduction

Morbidity and mortality caused by diabetes is of serious concern worldwide. Global prevalence of diabetes is expected to increase from 7.7% to 439 million by 2030 [1]. Diabetes is a disorder characterised by multiple defects at both the cellular and molecular levels in a wide variety of tissues. Resulting tissue damage in diabetes is associated with interactions and “crosstalk” between inflammatory and metabolic pathological processes such as endothelial dysfunction (via VCAM-1, ICAM-1, E-selection, vWF), procoagulation (PAI-1, fibronectin, and P-selectin), and inflammation (TNF*α*, IL-6, IL-1*β*, IL-18, and CRP) [2]. Cell-to-cell interactions, via gap junctional communication and connexon hemichannels, are now thought to play an important role in the molecular biology of diabetes. In this paper, we discuss current knowledge about connexins (Cxs) and their role in various aspects of diabetes.

## 2. Gap Junctions, Connexons, and Connexins

**Figure 1:**
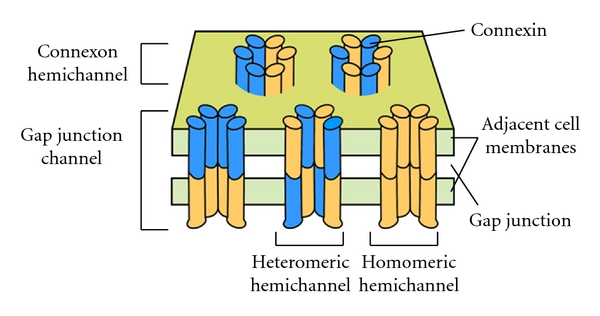
Multiple levels of gap junction channel structure. Individual transmembrane connexins (coloured blue and orange) assemble into hexamers called “connexon hemichannels.” Connexons from two adjacent cells join to make a channel across two plasma membranes (green), forming a “gap junction channel.”

Gap junctions are highly specialized structures, made up of channels spanning adjacent cell membranes, leaving a 2–4 nm extracellular “gap,” hence their name [3, 4]. In vertebrates, gap junction channels are assembled of trans-membrane proteins called “connexins.” Cxs are commonly described in terms of molecular mass (Cx43 represents the Cx protein of 43 kDa). Six Cx units oligomerize in the endoplasmic reticulum to form an annular hemichannel or “connexon.” Cxs can interact structurally in various ways, and connexons are regarded as homeric if the Cxs are of the same type or heteromeric if they are of different types. Similarly, the gap junctional channels may be formed by homeric or heteromeric connexons (see Figure 1). When the extracellular portion of a connexon docks with connexon from an opposing cell, a functional gap junction channel forms.

Tissue homeostasis is dependent on a complex system of cell communication over long distances by endocrine signalling, short distances via membrane signalling pathways (ion channels, G-protein coupled or tyrosine-kinase receptors), and by cell-to-cell contact. Gap junctions allow a unique form of communication; they mediate intracellular signals by directly connecting adjacent cells. Their purpose is to closely regulate the intracellular passage of ions, nutrients, metabolites, second messengers, and small biological molecules (up to 1000 Da). The multiplicity of isoforms and different structural combinations determine the biophysical permeation characteristics of a gap junction channel. Aside from forming gap junctions, the connexon hemichannels also provide a pathway for the release from cells of ATP, glutamate, NAD^+^, and prostaglandin E2, which act as paracrine messengers [5]. A dynamic cell-to-cell communication is advantageous as it avoids the transport mechanisms, ligand-receptor interactions, and dilutional effects associated with extracellular communication. A key function of gap junctions is sharing of metabolic demands across cells and tissues [6]. They are also of critical importance in cell differentiation, adhesion, and apoptosis, and they have regulatory roles in embryonic development, synaptic transmission, immune response, and carcinogenesis.

Cxs are now known to be multifaceted proteins that not only link to structural gap junctional complexes, but also to adhesion molecules, tight junctions, cytoskeletal elements, and cellular machinery, which facilitates their transport, assembly, function, and internalization. Initially, these gap junction protein complexes were termed the “Nexus” [7], which was the original structural name for the intracellular links. This term has come back into use, alongside the term “gap junction proteome” [8]. Once the gap junction channel assembly has nucleated and further binding partners are engaged, additional gap junction channels cluster and form “gap junction plaques.” Cxs are a homogenous family of proteins ubiquitously expressed in humans, and to date, 21 Cx genes have been reported in humans, and 20 in rodents.

## 3. Clinical Connexins

Since the first description of a mutation in the GJB1 gene coding for Cx32 in patients with the X-linked Charcot Marie tooth syndrome in 1993, disease-causing mutations have been detected in almost every Cx gene [9, 10]. Patients with the X-linked Charcot Marie tooth have an inherited progressive peripheral neuropathy, characterised by distal symmetrical neuropathy, muscle weakness, and sensory loss. Within myelinating Schwann cells, Cx32 has been localized to the paranodal regions and Schmidt-Lantermann incisures, which are found between the inner and outer layers of the coiled up cell. Connexon hemichannels composed of Cx32 therefore enable the rapid transport of small molecules and nutrients in a reflexive manner within individual Schwann cells. Although Cx32 is also found outside the peripheral nervous system in major organs like the liver, Cx32 mutations are associated only with X-linked Charcot Marie tooth [11].

In the sections below, we will outline the various Cxs associated with diabetes and its complications and discuss relevant studies (both animal and human) in the current literature.

### 3.1. Diabetes

Connexins are associated with the pathogenesis of both type I and type II diabetes (see Table 1) [12]. Cx36 forms the gap junctions between pancreatic beta cells which are required for normal basal insulin secretion and glucose-induced insulin release. Animal studies have shown that loss of Cx36 is associated with the loss of pulsatile insulin release, increase in basal insulin output, and reduced glucose-induced insulin release (these secretory defects are similar to those observed in type II diabetes) [13, 14]. The exact role of Cx36 in type II diabetes in humans is yet to be elicited. Cx36 is coded for by GJD2 gene, which is located on the 14q region of chromosome 15, a susceptibility locus for type II diabetes, and the diabetic syndrome [15]. Cx36 is a native protein of human pancreatic islets that mediates the coupling of beta cells through preferential exchange of cationic molecules and contributes to the control of beta-cell function by modulating gene expression [16]. Endogenous glucose production is a major determinant of fasting hyperglycaemia in type II diabetes [17]. Cx36 upregulation is associated with an increase in endogenous glucose production, predominantly of hepatic origin. Both Cx32 and Cx26 can form connexon hemichannels connecting liver hepatocytes. In animal studies, these connexon hemichannels are responsible for the regulation of blood glucose and hepatocyte glycogenolysis [18, 19]. Following electrical stimulation of sympathetic liver nerves, Cx32-deficient mice had inefficient mobilisation of glucose from hepatic glycogen stores [20]. The mechanism underlying this dysregulation in hepatocytes may involve interaction of Cxs with second messengers as Cx32-transfected HeLA cells had increased transmission of inositol 1,4,5-trisphosphate-mediated calcium waves to neighbouring cells [21].

Clinically, the complications of diabetes are vast. Due to the almost ubiquitous tissue distribution of Cxs, there has been interest in Cx changes in both diabetic macrovascular and microvascular disease.

### 3.2. Macrovascular Complications of Diabetes

Cardiovascular complications are the leading cause of morbidity and mortality in diabetes. Type II diabetes is an independent risk factor for major large vessel atherosclerosis. In this section, we will discuss the association of Cxs with atherosclerosis, dyslipidemia, peripheral vascular disease, coronary artery disease, hypertension, and arrhythmias.

Changes in the expression pattern of vascular Cxs (Cx37, Cx40, Cx43, and Cx45) have been observed in animal model studies during the formation of atherosclerotic plaques [22]. In atherosclerosis, Cxs may contribute to the inflammatory response through adhesion and transmigration of inflammatory cells. Cx37 is thought to function in monocyte adhesion and transmigration across the endothelium, whereas endothelial Cx40 is implicated in transmitting antiadhesive signals within the vascular endothelium [23].

The *in vivo* effect of diabetes on the expression pattern of vascular Cxs within major large vessels is not fully understood. The effects of statins on connexin expression in the arterial wall have been observed by Sheu et al. [24], who observed reduced expression of Cx43 in diabetic rat aortic walls and an increase in Cx43 levels following simvastatin treatment. In contrast, Hou et al. [25] found that in apoE-deficient mice, STZ-induced diabetes was associated with an increase in the burden of atheroma and downregulation of endothelial Cx37 and Cx40 outside the plaque areas. The downregulation of these endothelial Cxs was exacerbated by short-term treatment with simvastatin. This study also raises the issue of how Cx expression is altered in dyslipidemia.

In dyslipidemia, there is a reduction in Cx37 and Cx40 in vascular endothelial cells and increase in Cx43 expression in both vascular endothelial cells and smooth muscle cells. This is supported by both hyperlipidemic mice studies by Yeh et al. [26] who found reduced expression of endothelial Cx37 and Cx40 and human observational data. An association between Cx37 polymorphism and peripheral arterial disease in patients with type II diabetes has been observed [27]. Animal studies have also shown that treatment of dyslipidemia reduces Cx levels. Dlugosová et al. [28] identified Cx43 in the aorta (in both the tunica media and to a lesser degree in the endothelium) of adult hereditary hypertriglyceridemia rats, and that treatment with omega-3 polyunsaturated fatty acids and atorvastatin markedly lowered Cx43 expression.

Atherosclerosis causes coronary arteries to progressively stenose, and despite the development of drug-eluting stents, restenosis is a serious clinical problem associated with significant morbidity and mortality. Diabetes still remains one of the most important risk factors for restenosis following stent implantation. The levels of Cx43 are increased in the smooth muscle cells of atherosclerotic arteries of both humans and mice [29–31]. Recent study of connexins following balloon angioplasty has shown that this process is accompanied by elevated expression of Cx43 in endothelial cells and SMC of the media and later the neointima [32, 33]. Neointima formation following balloon catheter injury is significantly reduced in heterozygous Cx43 knockout mice (Cx43^+/−^), suggesting a correlation between neointima formation and high levels of Cx43 during the inflammatory response to injury [34].

In humans, Type II diabetes is associated with hypertension, and animal studies have demonstrated that multiple Cx changes are associated with the remodelling of the arterial wall that occurs in response to hypertension. These Cx changes are complex, and various expression patterns are described in different models of hypertension [35]. Renin secretion is regulated by coordinated signalling between the various cells of the juxtaglomerular apparatus and is dependent on Cx40. Cx40 knockout (Cx40^−/−^) mice are initially found to be hypertensive [36]. Further studies [37] using Cx40^−/−^ mice have shown that chronic hypertension upregulates Cx40 in endothelial cells, but Cx37, Cx43, and Cx45 in smooth muscle cells. It is hypothesized that for Cx37, Cx40, and Cx45, control of expression does not depend on circulating plasma levels of renin and angiotensin II, but these hormones are required for selective expression of Cx43 which may occur through angiotensin II activation of the extracellular signal-regulated kinase and NF-*κ*B pathways.

As gap junctions mediate the cell-to-cell propagation of current flow that govern orderly contraction of the healthy heart, there has been considerable investigation into the role of Cxs (Cx40, Cx43, and Cx45) in arrhythmic diabetic heart disease [38]. In rat studies of STZ-induced diabetes, there is associated structural remodelling of Cxs (Cx40, Cx43, and Cx45) in the sinoatrial node and ventricular myocytes, which may partially account for sinus arrhythmias, ventricular arrhythmias, and prolonged QT/QRS conditions found in human diabetes [39, 40]. Inoguchi et al. [41] investigated the underlying mechanisms by which Cx43 is involved in generation of arrhythmias in diabetic rat ventricular myocytes. Increased levels of phosphorylated Cx43 were normalized by use of a protein kinase C *β*-specific inhibitor. The mechanism proposed was that hyperglycaemia causes PKC activation, which in turn causes phosphorylation of Cx43 and impaired ventricular contraction. Associations between Cx43 and dilated cardiomyopathy have also been identified. Mutations in Lamin A/C gene (*LMNA*) causing dilated cardiomyopathy are related to a decrease in Cx43 levels [42]. In these conditions, it is thought that the altered distribution of Cxs (such as Cx43) and consequent interruption of gap junctional communication within the myocardium lead to decreased velocity of electrical conduction, generation of reentry arrhythmias, and arrhythmias causing sudden death [43].

### 3.3. Microvascular Complications of Diabetes

#### 3.3.1. Nephropathy

 Diabetic nephropathy is the commonest cause of end-stage renal disease. In total, nine Cxs have been found in the kidney (Cx26, Cx30.3, Cx31, Cx32, Cx37, Cx40, Cx43, Cx45, and Cx46) [44–46]. In the preglomerular vessels, cell-to-cell communication has an important physiological role in the functional coupling between adjacent nephrons supplied by a single interlobular artery. In rat preglomerular renal microvasculature, there is extensive expression of Cx37, 40, and 43 mRNA in endothelial cells and Cx37 mRNA in smooth muscle cells [47]. In the postglomerular renal microvasculature, only Cx43 is found within the endothelial cells [44].

In diabetic nephropathy, early functional changes occur in the nephron at the level of the glomerulus including glomerular hyperfiltration and hyperperfusion, before the onset of measurable clinical change. Over time, further structural changes like thickening of the glomerular basement membrane, glomerular hypertrophy, and mesangial expansion take place [48]. In animal models of diabetes, changes in Cxs are associated with these key pathophysiological features.

Glomerular hyperfiltration in early diabetic nephropathy results from deceased resistance in both afferent and efferent arterioles of the glomerulus. With the development of early diabetic nephropathy in STZ rats, Hillis [44] found that levels of Cx40 were increased in smooth muscle cells of the preglomerular arterioles, while levels of Cx43 were decreased in endothelial cells of the postglomerular arterioles. This suggests that changes in these particular Cxs are associated with impaired autoregulation and subsequent glomerular hyperfiltration. As discussed in Section 3.2 above, upregulation of Cx40 in preglomerular vessels is of pathophysiological significance in animal models of hypertension, and normal blood-pressure-controlled release of renin is dependent on Cx40. The association between changes in Cxs and glomerular hyperfiltration has also been studied in Zucker diabetic fatty (ZDF) rats. Takenaka et al. [49] found that compared to control rats, ZDF rats had glomerular hyperfiltration. These features were associated with reduced Cx37 in renin secreting cells and enhanced phosphorylation of Cx43 (an event that interfered with gap junction functioning). Aside from the renal microvascular cells, glomerular mesangial cells, which are normally found around the renal blood vessels, are involved in the pathogenesis of diabetic nephropathy. The studies in diabetic rats by Hillis [44] also showed increased Cx37, Cx40 and absence of Cx43 in both extra- and intramesangial cells. Later studies by this group [50] showed that high glucose concentrations downregulated Cx43 and promoted senescence of the glomerular mesangial cells.

Changes in Cxs in human kidney cells have been studied in culture. Not all of the cell types have been studied, and potential clinical applications require more consideration. An interesting possibility is that Cx43 has a protective effect preventing renal damage. Hills et al. [51] studied human collecting duct cell lines and observed that as glucose concentrations increased, there was a time-dependent increase in the levels of Cx43. The increase in Cx43 and gap junctional communication in response to high glucose was correlated with functional acceleration of calcium transients between cells. It was concluded that Cx43 may protect the collecting duct from damage associated with established diabetic nephropathy. Furthermore, levels of Cx43 have also been studied in human diabetic nephropathy as a “predictive marker” of disease progression and severity. Sawai et al. [52] found that downregulation of Cx43 within podocytes was closely associated with disease progression in established diabetic nephropathy and correlated with the degree of future decline in renal function.

#### 3.3.2. Retinopathy

 Throughout the human retina, numerous Cxs have been studied [53, 54]. Abnormalities in Cx have been linked to the development of human diabetic retinopathy. In human pericyte cell culture studies by Li et al. [55], high glucose concentrations directly induced downregulation of Cx43 expression and inhibition of gap junctional intracellular communication in retinal pericytes. Accelerated apoptosis of retinal vascular cells is a key feature of diabetic microangiopathy and is caused by inadequate transportation of necessary cell survival and signaling molecules. Studies of retinal microvascular endothelial cells have found that decreasing Cx43 levels leads to a significant increase in the occurrence of apoptosis [56]. It has also been shown that high-glucose-induced oxidative stress hyperphosphorylates Cx43, leading to the disassembly of gap junctions and degradation of Cx43 through the proteasome pathway [57, 58]. Diabetic retinopathy also is associated with accelerated apoptosis in retinal mitochondria. In the retinal mitochondria, it has been shown that matrix metalloproteinase-2 activation modulates the abundance of heat shock protein-60. This is thought to then lead to opening of the mitochondrial transition pores through disruption of Cx43 [59].

#### 3.3.3. Erectile and Bladder Dysfunction

 Patients with diabetes have a higher incidence of erectile dysfunction and bladder dysfunction, and both these conditions are characterised by early onset and increased severity. The role of Cxs in the development of erectile dysfunction and higher bladder detrusor muscle activity in diabetes is emerging through recent animal studies. Suadicani et al. [60] found markedly decreased expression of Cx43 in the penile corpora and increased expression of Cx43 in the urinary bladder following induction of diabetes in STZ rats. There is now evidence that nitric-oxide-dependent mechanisms alone are not sufficient for maintaining normal erectile function, suggesting that other processes such as gap junctional communication facilitate erectile function by enabling passage of ions and small molecules necessary for the relaxation of corporal smooth muscle cells [61]. In bladder detrusor muscle overactivity, in addition to hemichannel paracrine signalling mechanisms via ATP activation of purinergic receptors, other potential mechanisms that could be responsible for bladder dysfunction include transcriptional regulation of Cx43 by basic fibroblast growth factor (bFGF), and signaling via the ERK-AP-1 pathway [62].

#### 3.3.4. Peripheral Neuropathy

 Diabetic peripheral neuropathy involves both somatic and autonomic nerves, causing sensory deficits, palsies, and cardiovascular, gastrointestinal, and genitourinary dysfunction. The initiation of diabetic peripheral neuropathy is not well understood. Various mechanisms are involved in the pathogenesis of this condition, including terminal nerve destruction, decreased neuronal conductance, demyelination, microvascular insufficiency, inflammation, and oxidative damage. As discussed above, Cx32 gene mutation in humans is strongly associated with peripheral neuropathy in X-linked Charcot Marie tooth disease. It is also known that gap junctional communication is important in the maintenance of the perineurium blood-nerve barrier. Reduced levels of Cx32 and 26 have been observed in the perineurium in STZ rat model of diabetes-related peripheral neuropathy and are likely to contribute to this condition [63].

#### 3.3.5. Chronic Ulceration

Diabetic patients are more prone to develop foot ulcers, for several reasons including neuropathy, vascular disease, and foot deformities [64]. Diabetic foot wounds are very slow to heal [65], and numerous dysfunctional events occur in all phases of diabetic wound healing (acute inflammation, proliferation, and remodelling). There is impairment of the inflammatory response, macrophage function, cytokine, chemokine and growth factor production, extracellular matrix production, angiogenesis, keratinocyte, and fibroblast migration and reepithelialisation [66].

At least ten of the Cxs are found within the skin (Cx 26, 30, 30.3, 31.1, 32, 37, 40, 43, and 45) [67]. However, the spatial and differential expression pattern of Cxs in the skin does vary between animals and humans [68]. In humans, Cx43 is the most abundant Cx of the epidermis, expressed throughout the spinous, granular, and basal cell layers, sebaceous glands, hair follicles, and blood vessels [69]. Cx26 is less abundant in the epidermis and is found in a patchy distribution in the basal keratinocytes, but is highly expressed in hair follicles and sweat glands [70]. In animal models, when diabetes is induced, there are significantly altered Cx levels in various layers of the skin in response to injury. Studies by Wang et al. [71] demonstrated that in the skin of rats with STZ-induced diabetes within two weeks of the onset of diabetes, Cx43 and Cx26 proteins were significantly downregulated in the epidermis, whereas Cx43 was upregulated in the dermis. These findings indicate that diabetes does not uniformly repress connexin expression throughout the layers of the skin, but can also enhance it. Furthermore, after wounding in diabetic rats, the wound edge keratinocytes upregulate Cx43 in the first 24 hours after injury. The keratinocytes then form a thickened bulb of nonmigrating cells at the wound margin and do not start migrating until Cx43 downregulates, around 48 hours after wounding. This increased level of Cx43 then delays reepithelialisation and the wound-healing process. Preventing the abnormal expression of Cx43 in wound edge keratinocytes rescued wound healing to normal rates or even better [70]. If Cx43 expression in human diabetic wounds is altered in a similar way to the STZ diabetic rat model, targeting Cx43 may provide a new clinical application for the treatment of diabetic ulceration. Qiu et al. [72] initially studied the topical application of a Cx43 antisense olidodeoxynucleotide (Cx43 asODN) pluronic gel to murine wounds. This treatment had a rapid effect, noticeable as early as six hours after injury. The treated wounds appeared less erythematous and oedematous, with reduced exudate and less gape than control wounds. In diabetic STZ rats, Kamibayashi et al. [70] also found that the application of Cx43 asODN pluronic gel inhibited the upregulation of Cx43. This treatment significantly improved wound healing, compared to control wounds, doubling the rate of reepithelialisation.

In human diabetic ulcer biopsies, Brandner et al. [73] has reported persistence of Cx43 expression in wound-edge keratinocytes. Other studies of human diabetic fibroblasts have also shown increased gap junctional communication, which correlated with reduced cell proliferation [74]. Overall, Cxs are associated with tissue response to injury directly, via gap junctional communication, and may also contribute to the wound repair process indirectly [75]. The downregulation of Cx43 may have direct effect on the migratory ability of the cells involved in wound healing, through its central role in the protein “nexus.” Cx43 downregulation may also exert an influence at the transcriptional level on numerous other genes and proteins [76].

## 4. Conclusion

The study of Cxs in diabetes is of interest. The changes in Cxs found in diabetes are associated with both direct effects within the vasculature and indirect effects, by impairment of homeostasis in vital organs such as the liver and kidney. It is unlikely that targeting individual connexins will cure diabetes. However, it may be possible to alleviate some of the symptoms of microvascular complications, as demonstrated in recent work using topical Cx43 asODN gel treatment. As considered in nephropathy studies, Cxs may also be used as future predictors of both diabetes progression and severity.

## Figures and Tables

**Table 1:** Connexin changes associated with diabetic complications (upward and downward arrows indicate increase or decrease in Cx levels, VECs: vascular endothelial cells, SMCs: smooth muscle cells).

Diabetic complication	Connexin changes associated with diabetes	Type of study	References
Macrovascular			
Atherosclerosis	Early atheroma **↓**Cx37 VECs and circulating monocytes	Human and animal diabetic studies	[22]
	Advanced atheroma **↑**Cx37 SMCs, ↓Cx40 VECs		
	Early atheroma **↑**then↓Cx43 SMCs		
	Advanced atheroma **↑**Cx37, **↑**Cx43 macrophage foam cells		
Dyslipidemia	↓Cx37, ↓Cx40 VECs	Human and animal diabetic STZ rat studies	[27, 28]
	**↑**Cx43 VECs and SMCs		
Hypertension	**↑**Cx40 VECs	Animal studies (e.g. Cx40^−/−^ mice)	[35–37]
	**↑**Cx37, 43, 45 SMCs		
Arrhythmias	Ventricular myocytes **↑**Cx43 (both total and phosphorylated forms)	Animal diabetic STZ rat studies	[39–41, 43]
	Atrial myocytes (**↑**Cx43 but ↓phosphorylated form)		
	Sinoatrial node **↑**Cx45>**↑**Cx43 and Cx45		

Microvascular			
Nephropathy	↓Cx37 renin secreting cells	Animal diabetic STZ and ZDF rat studies	[44–49]
	**↑**Cx40 SMCs preglomerular arterioles		
	↓Cx43 SMC postglomerular arterioles and mesangial cells		
	**↑**Cx43 collecting ducts, podocytes	Human diabetic studies	[50, 51]
Retinopathy	↓Cx43 retinal pericytes, microvascular endothelial cells	Human diabetic cell studies	[54–58]
Erectile dysfunction Detrusor overactivity	↓Cx43 penile corpora **↑**Cx43 bladder detrusor muscle	Animal STZ diabetic rat studies	[59–61]
Peripheral neuropathy	**↑**Cx26, **↑**Cx32 perineurium	Animal diabetic STZ rat studies	[62]
Wound healing	↓Cx43, ↓Cx26 epidermis	Animal diabetic STZ rat studies	[70, 71]
	**↑**Cx43 dermis, wound edge keratinocytes first 24 hours after injury		
	**↑**Cx43 wound edge keratinocytes	Human studies	[72]
